# Prenatal body fluid analysis in the evaluation of CAKUT

**DOI:** 10.1007/s00467-026-07375-7

**Published:** 2026-06-05

**Authors:** Thomas Simon, Bénédicte Buffin-Meyer, Julie Klein, Stéphane Decramer, Joost P. Schanstra

**Affiliations:** 1 South-West Reference Center for Rare Diseases (SORARE), Toulouse, France; 2 Pediatric Nephrology Department, Toulouse Children’s Academic Hospital, Toulouse, France; 3 Institute of Cardiovascular and Metabolic Disease, Institut National de La Santé Et de La Recherche Médicale (INSERM), Toulouse, 31432 France; 4 Toulouse University, Toulouse, France

**Keywords:** Prenatal diagnosis, Amniotic fluid, Fetal urine, Prognostic biomarkers, Peptides, Mass-spectrometry, Clinical implementation

## Abstract

Congenital anomalies of the kidney and urinary tract (CAKUT) are the leading cause of chronic kidney disease in children. Although prenatal ultrasound enables early detection, its ability to predict postnatal renal outcome, particularly in bilateral cases, remains limited due to substantial phenotypic variability and the inability of imaging to capture ongoing disease processes. Genetic testing improves etiological classification but shows weak genotype–phenotype correlations and limited prognostic value, reflecting the multifactorial nature of CAKUT and prompting investigation of molecular biomarkers in prenatal body fluids. This review summarizes current prenatal prognostic strategies in CAKUT, including imaging, genetics, and downstream molecular approaches such as transcriptomics, metabolomics, and proteomics, with particular emphasis on peptide-based profiling. Among these, prenatal peptide signatures derived from fetal urine or amniotic fluid have demonstrated strong prognostic performance for predicting postnatal renal outcome and may capture early molecular processes related to tissue remodeling and inflammation. However, translation into clinical practice remains challenging. Validation requires large prospective multicenter studies that are difficult to conduct in rare diseases and often lack sustained funding. In addition, implementation will likely rely on mass spectrometry-based assays in clinical laboratories. Despite these hurdles, integrating molecular peptide profiling with improved and standardized imaging approaches may enhance prenatal counselling and clinical decision-making in CAKUT.

**Graphical abstract:**

**Figure:**
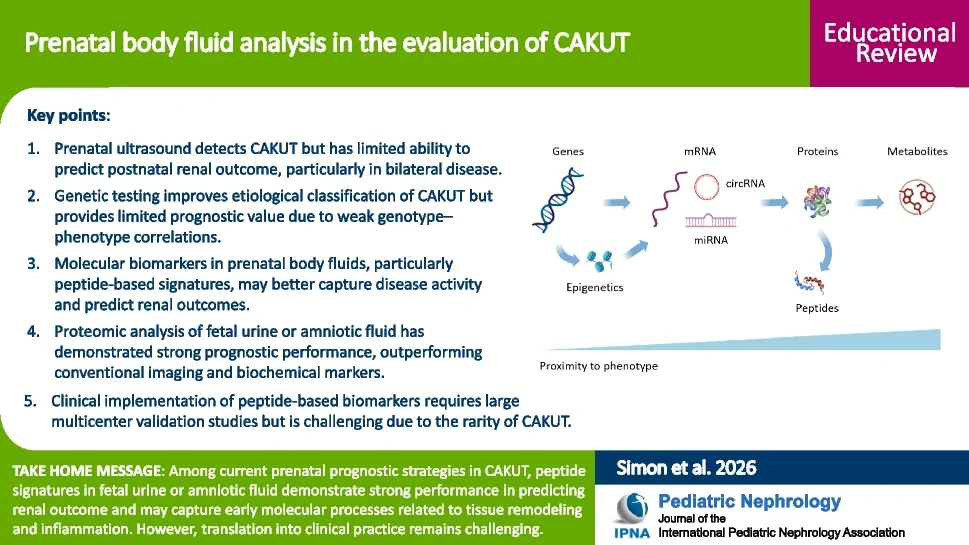
A higher resolution version of the Graphical abstract is available as Supplementary information

**Supplementary information:**

The online version contains supplementary material available at https://doi.org/10.1007/s00467-026-07375-7.

## Introduction

Congenital anomalies of the kidney and the urinary tract (CAKUT) are characterized by defects in embryonic kidney development. CAKUT are among the most frequent congenital anomalies with a prevalence of 3–6/1000 births [1]. Usually detected at an early stage by ultrasound during pregnancy, they are the predominant cause of chronic kidney disease (CKD) in children [2] and account for ~ 50% of pediatric and young adult stage 5 chronic kidney disease (CKD 5) cases [3]. CAKUT include ureteropelvic junction (UPJ) obstruction, multicystic dysplastic kidneys (MCDK), kidney agenesis, kidney hypoplasia, kidney dysplasia, vesicoureteral reflux (VUR), megaureter, ectopic ureter, horseshoe kidney, duplex collecting system, and posterior urethral valves (PUV). Nephrogenesis is a spatiotemporal process, and the diversity of CAKUT etiologies reflects disruptions occurring at different stages of embryonic kidney development. Early developmental defects typically result in severe anomalies such as renal agenesis or malformations, whereas disturbances arising later in gestation more commonly lead to abnormalities of the ureter and collecting system [4]. However, this distinction is not absolute since certain collecting system abnormalities, such as VUR, may originate from early developmental events, including ectopic ureteric bud outgrowth.

Unilateral lesions, even anatomically severe, typically do not result in a severe functional phenotype due to compensation by the contralateral kidney. However, with the exception of isolated lower urinary tract obstruction (LUTO) without kidney parenchymal lesions, a prenatal bilateral presentation is associated with a severe kidney outcome [5]. In extreme cases (bilateral agenesis or MCDK), it leads to early and sustained anhydramnios which is strongly associated with pulmonary hypoplasia and worsens overall neonatal outcome [5]. Despite the major impact of CAKUT on pediatric CKD, the clinical management of prenatally detected bilateral CAKUT has changed little over the last decades and relies mostly on imaging techniques. In this review, we propose that optimal management of CAKUT pregnancies will require moving beyond imaging alone toward an integrated framework that combines imaging findings with molecular profiling of prenatal body fluids.

## Current prenatal strategies

### Imaging in CAKUT

The detection of CAKUT in utero has substantially improved with the wide-spread use of prenatal ultrasound after its introduction about 40 years ago. However, following ultrasound-guided detection of CAKUT, the obstetrician is left facing a large variability in phenotype and disease outcomes [6]. The prenatal sonographic appearance, except in extreme cases (e.g., bilateral agenesis and anamnios), does not faithfully predict the severity of the disease and its progression [7]. The low predictive value of ultrasound probably has several origins. Ultrasound only allows a macroscopic assessment of kidney parenchyma and urinary tract [8] and not the evaluation of the active ongoing disease process. Differences in equipment, protocols, and interpretation by clinicians lead to substantial inter-observer variations [9]. Amniotic fluid (AF) volume is frequently used as a proxy for renal function, but its estimation by ultrasound remains unsatisfactory, especially with abnormal AF volumes (oligo- and polyhydramnios) [9]. This low predictive value of ultrasound has led to situations where postnatal renal function of bilateral CAKUT proved to be normal while termination of pregnancy (TOP) was considered (but not performed) [10]. Conversely, there have been cases in which TOP was performed but fetopathology concluded that the kidney ultrastructure seems compatible with normal life [11]. However, the value of imaging for prenatal outcome prediction is likely to improve further. Proof-of-concept studies have demonstrated that combining multiple prenatal ultrasound features within a standardized ultrasound-based assessment protocol enhances the prediction of postnatal kidney function compared with the use of AF volume alone [12]. Such standardization is expected to benefit from further advances through artificial intelligence (AI)-based analysis of ultrasound data. For example, Miguel et al., showed that AI approaches can discriminate between healthy fetuses and those with multicystic dysplastic kidney (MCDK) or urinary tract dilatation [13]. AI-based analysis is anticipated to reduce operator-dependent variability and enable the detection of subtle patterns and temporal changes that may not be readily identifiable by the human eye [14]. A key limitation of AI lies in its reliance on “black-box” models, where the underlying decision-making process is not readily interpretable; however, this limitation can be mitigated through the use of explainable AI (xAI), which aims to enhance transparency by providing interpretable insights into the factors driving predictions, decisions, or classifications [13].

Magnetic resonance imaging (MRI) can provide complementary information in the evaluation of CAKUT, particularly by improving morphological characterization and occasionally identifying anomalies that may not be detected by ultrasound. However, ultrasound remains the primary diagnostic modality in prenatal practice. Although MRI techniques have shown promise for the non-invasive assessment of renal function in several kidney diseases in adults and children, their application in the fetal setting remains limited. The potential role of MRI in evaluating fetal renal function has not yet been extensively investigated and still requires further validation. Consequently, fetal MRI is not currently used in routine clinical practice for the assessment of fetal renal function [15].

Thus, although instrumental in the prenatal detection and pending future improvements through standardized protocols and the use of xAI and/or MRI, ultrasound has limitations as a tool to assess the severity and the risk for progression of CAKUT pregnancies. Modern strategies based on molecular assessment of disease could help to overcome these limitations.

### Genetic risk factors in CAKUT

Unsurprisingly, as in many rare diseases, clinically focused research on CAKUT molecular dysregulations has largely concentrated on the identification of gene anomalies, although experimental models in translational strategies have also contributed substantially to our understanding of disease mechanisms [16]. Next-generation sequencing-based genetic testing has helped to determine the underlying genetic cause of many diseases. This has contributed to recurrence-risk estimation and family planning. Currently, approximately 54 genes related to CAKUT have been identified. While *PAX2* and *HNF1B* remain among the best-characterized and most frequently reported genes, several additional genes with genetic and functional evidence, including *TBX18*, *EYA1*, *SIX1*, *ROBO2*, and *GATA3* have been implicated in monogenic or syndromic forms of CAKUT, reflecting the complexity of nephrogenesis and ureteric tract development [17]. Limited genotype–phenotype correlations have been described. We have recently shown in a large cohort (more than 500 patients) in the context of the European rare disease consortium ERKNet, that in patients with *HNF1B* disease, the progression toward CKD stage 3 was significantly delayed in patients with the 17q12del compared to patients with *HNF1B* variants. Furthermore, in the latter, the progression depended on the specific domain where the variant was located [18]. Finally, copy-number variants (CNVs) have also been described in CAKUT and suggest that ~ 10% of cases are caused by CNVs in patients [19]. There is some evidence in PUV that copy-number variants (CNVs > 100 kb) may predict postnatal renal function. Faure et al., observed in a cohort of 45 children with PUV that renal function at 1 year of age was approximately 50% worse in patients with CNVs than those without CNVs [20]. Although promising, these observations have not been confirmed to date.

Combinations (i.e., signatures) of pathogenic molecular variants in known CAKUT genes have not yet been explored for their predictive value in CAKUT. This is due to the fact that many of the identified mutations are rare or limited to single patients/families, thereby limiting their recurrence and their validation across cohorts. In addition, more recently, next-generation sequencing studies have modified these risk estimates and prevalence of genes previously marked to contribute to CAKUT incidence. For example, the major suspects, *PAX2* and *HNF1B* variants or deletions, were found only in 0.7% and 0.3% (versus ~ 15% in previous studies [1]), respectively, of all 453 CAKUT patients in one study [21]. The low frequency of CAKUT-associated gene variants and deletions therefore limits the ability to investigate their combined effects in a robust manner. It thus appears that prenatal genome-based prognostics for CAKUT is currently mostly at a standstill with little perspective for progression.

## Impact of environment in CAKUT development

**Fig. 1:**
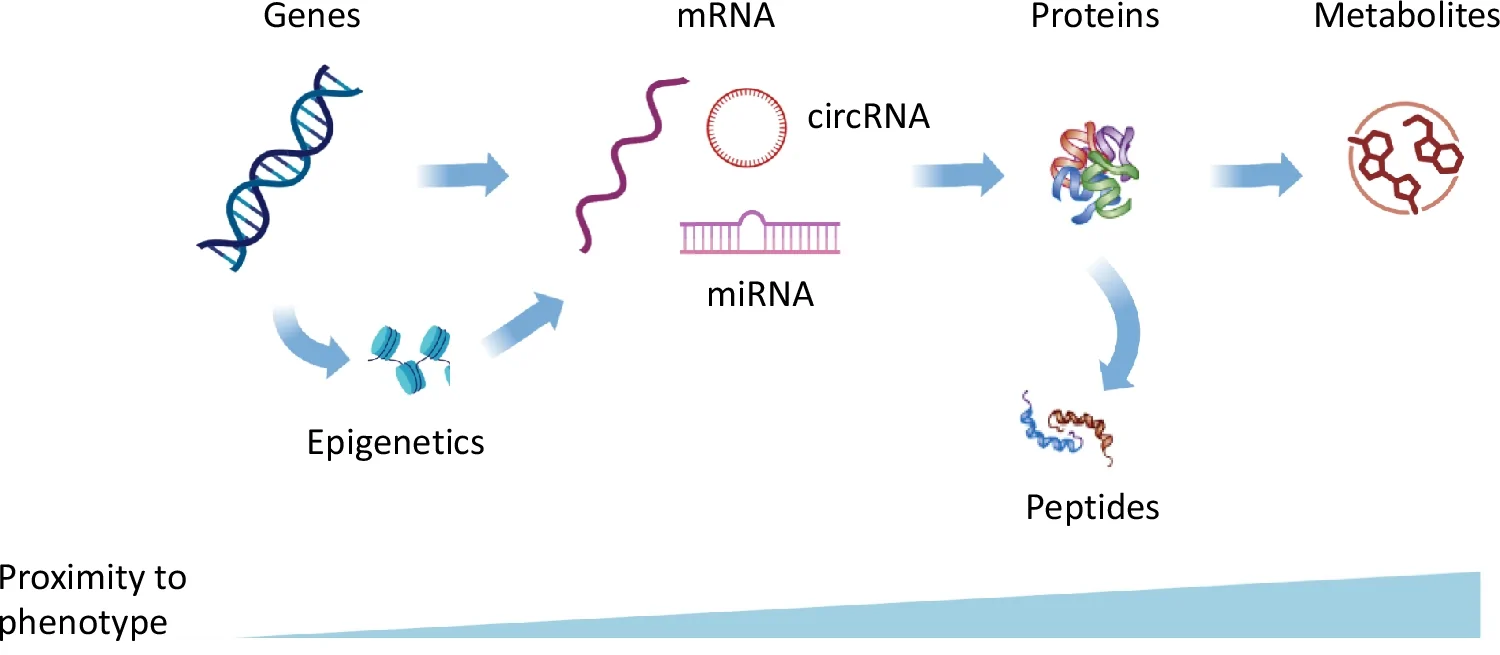
Conceptual overview of molecular layers linking genotype to phenotype. Biological information flows from genes and epigenetic regulation to RNA species (mRNA, miRNA, circRNA), proteins and peptides, and ultimately metabolites. Transcriptomics provides information closer to phenotype than DNA but mRNA abundance only approximates effective protein concentration and activity. In body fluids, mRNA is highly susceptible to degradation, whereas non-coding RNAs such as miRNAs and circRNAs are more stable and have therefore been explored as potential biomarkers. Proteins and peptides reflect both acute and long-term biological processes. Metabolites represent the most downstream molecular layer and can closely reflect the current phenotype, although their levels are strongly influenced by environmental and physiological variability. The lower panel illustrates the increasing proximity to phenotype from genes to metabolites. Adapted from Klein et al., Kidney International, 2016 [24], with permission. Other images were generated using Biorender

This clear absence of a geno- to phenotype correlation points to the potential influence of environmental factors in CAKUT and suggests that its pathogenesis is multifactorial. Environmental factors currently identified to be associated with CAKUT include pre-gestational maternal diabetes mellitus, maternal overweight/obesity, and drug use [1]. More recently, assisted reproductive technologies have also been associated with increased occurrence of CAKUT [22]. These environmental factors can impact gene expression via epigenetic changes, including DNA methylation and histone modification (Fig. 1), potentially causing forms of CAKUT, as demonstrated in mice [23].

The combination of underlying gene anomalies and the impact of different environmental factors at different points in time will have a variable impact and explains, at least in part, the wide phenotypic variability and severities observed in CAKUT.

## Downstream from the genotype

Thus, although CAKUT is detected increasingly early, in some cases accompanied by the knowledge of the underlying genetic anomaly, the multifactorial character of CAKUT hampers the ability to faithfully predict the severity of the disease and its progression. This multifactorial character of CAKUT forces us to look for molecular traits that can measure the CAKUT dynamics. In contrast to the genome, traits downstream of the genome (e.g., coding and non-coding RNA, proteins, peptides, and metabolites) in prenatal body fluids are more susceptible to capture the environmental impact since those traits are closer to the phenotype (Fig. 1). Furthermore, combination of these traits in the form of molecular signatures will potentially allow us to grasp the dynamics of the disease by detailed insight into pathway(s) activation.

### Coding and non-coding RNA and metabolites in prenatal body fluids

Assessment of global RNA abundance (transcriptomics) is closer to the phenotype than DNA and may better reflect disease activity; however, mRNA levels remain only a proxy for effective protein concentration and function [25]. Transcriptome analyses capture both coding mRNAs and non-coding RNAs, including microRNAs (miRNAs) and circular RNAs (circRNAs). In practice, mRNA has limited utility as a biomarker in body fluids due to variable exoribonuclease-mediated degradation, although transcriptome analysis of AF has allowed researchers to obtain insight into the molecular changes in, e.g., gestational age or preterm birth without direct clinical application [26]. By contrast, miRNAs are relatively stable in body fluids such as urine and have therefore attracted interest as non-invasive biomarkers of kidney disease [27]. Experimental data support a role for miRNA pathways in renal development, and altered miRNA abundance has been reported in CAKUT tissues, with small studies suggesting differences in urinary profiles in obstructive uropathies. However, these studies remain limited, and their predictive value and clinical utility are not yet established; methodological challenges further complicate their use [27]. CircRNAs, owing to their resistance to degradation, are also stable and have shown biomarker potential in other fields such as oncology, but evidence in CAKUT is currently lacking [27]. Overall, the stability of miRNAs and circRNAs makes them attractive candidates for prenatal biomarkers, although this potential remains largely unexplored.

Metabolites, as downstream products of genomic, transcriptomic, and proteomic processes, provide a close representation of the phenotype. Proof-of-principle studies support their potential in CAKUT, including urinary metabolites in ureteropelvic junction obstruction [28] and fetal urine in PUV [29]. However, the metabolome is highly sensitive to biological and environmental variability (e.g., age, diet, medications, circadian and seasonal factors), as well as pre-analytical conditions such as sample handling and storage [30]. While standardization can mitigate some of these effects, residual variability complicates biomarker discovery and interpretation. Nonetheless, advances indicate that disease-related signals can be distinguished from confounding influences such as diet [31]. Although AF may be relatively protected from external variability, the field remains underdeveloped and requires larger, well-controlled studies to establish robustness and clinical relevance.

### Analysis of proteins and peptides in (prenatal) body fluids

As stated above, among the body fluid-based omics traits that could provide prognostic information in CAKUT, proteins appear to reconcile representation of the phenotype, stability, availability, and accessibility. Proteins can present both acute (e.g., changes in circulatory cytokine levels) and long-term (e.g., extracellular matrix (ECM) accumulation) events. In addition, proteins are the major drug targets.

**Fig. 2:**
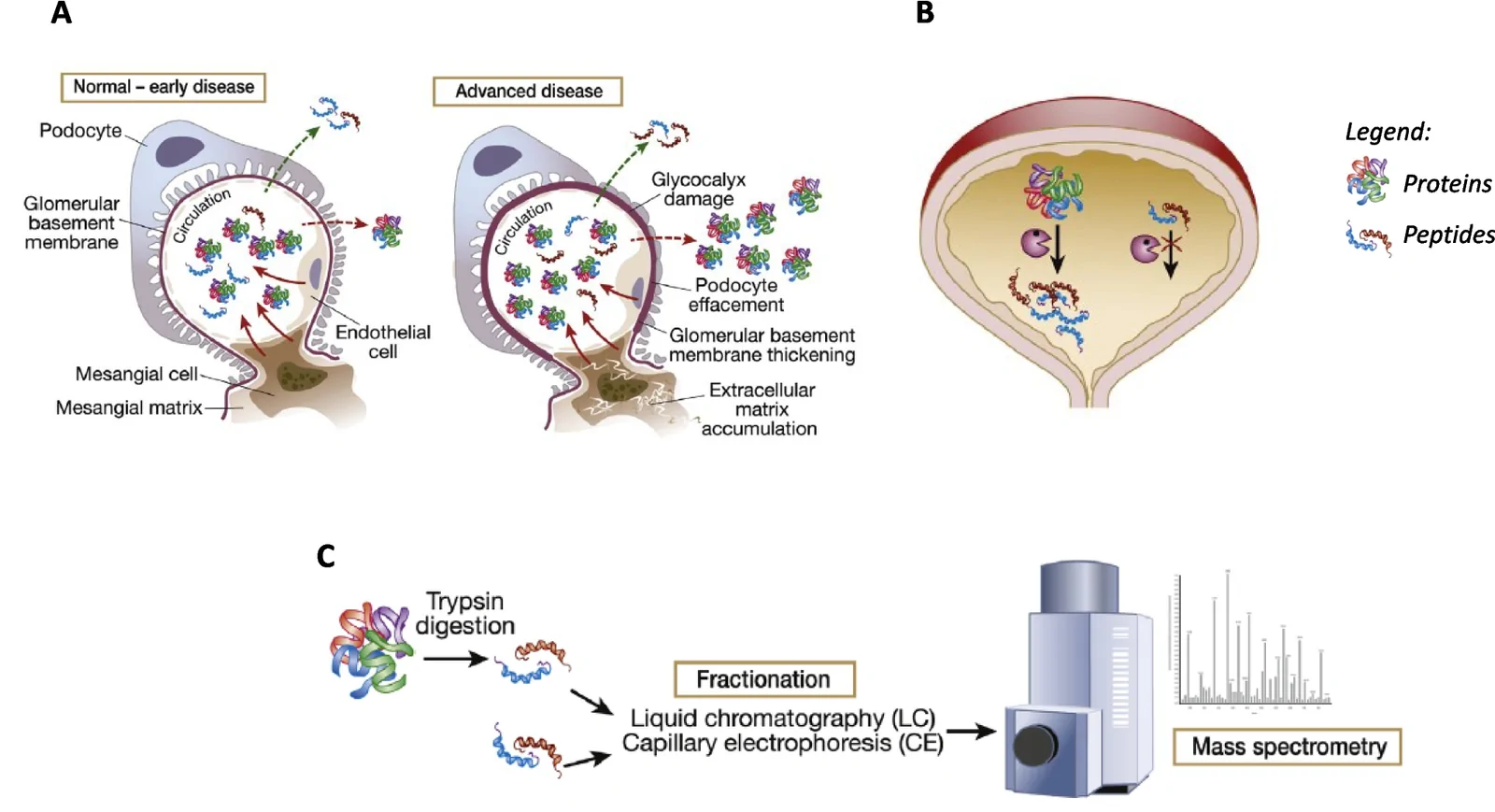
Proteins versus peptides of biomarkers of (kidney) disease. **A** Schematic representation of glomerular filtration in normal/early disease and advanced disease. Under physiological conditions, endogenous peptides generated from protein degradation can pass the intact glomerular filtration barrier and appear in urine, enabling early detection of disease-related changes. In contrast, most full-length proteins are retained and become detectable in urine mainly after glomerular filtration barrier damage. **B** Endogenous peptides in urine and AF are relatively stable degradation products and remain largely unchanged after sample collection, supporting their use as reliable biomarkers. **C** Comparison of analytical workflows. Peptidomic analysis can directly measure endogenous peptides by liquid chromatography or capillary electrophoresis coupled to mass spectrometry, whereas proteomic analysis of full-length proteins typically requires additional steps such as tryptic digestion and fractionation, introducing additional analytical variability. Adapted from Klein et al., Kidney International, 2016 [24], with permission

Both full-length proteins and protein degradation products, i.e., endogenous peptides with molecular weights up to 15–20 kDa, appear to be suitable as biomarkers of disease, with full-length proteins potentially more informative on the pathophysiology of disease [24, 32]. The potential reasons for the potential higher applicability of peptides relies on the fact that: (i) peptides are filtered and excreted by the kidney under physiological conditions, and changes in their urinary abundance can be detected at an early stage of the disease where the glomerular filtration barrier is still functional. Proteins are for the most part detected in urine only after glomerular filtration failure (Fig. 2A); (ii) the stability of the urinary and AF peptides is much higher than proteins; peptides being essentially degradation products less amendable to further cleavage. Once collected, peptides also remain very stable, and urine can be stored for 3 days at 4 °C and 6 h at room temperature without significant changes in the urinary peptidome (Fig. 2B). Similar observations hold for AF peptides; and (iii) proteome or peptidome analysis is, in the majority of cases, performed by mass spectrometry (MS)-based strategies, and the analysis of the endogenous peptide body fluid content is also more reproducible than full-length protein analysis since it can be performed without tryptic digestion. In contrast, MS analysis of full-length proteins usually needs tryptic digestion requiring additional analytical steps (purification, precipitation) leading to the introduction of additional variability (Fig. 2C). Robust body fluid biomarkers with clinical value are thus more likely to be present among the peptides than among the proteins.

In the remainder of this review, we will focus on employing protein-focused prenatal body-fluid strategies in CAKUT and discuss how they could contribute to improved management. We will also discuss the implementation of such strategies in routine clinical management of CAKUT and the obstacles still to overcome.

## Clinical proteomics in CAKUT (Table 1)

**Table 1:** Studies using prenatal body fluid protein-based markers in CAKUT

Body fluid	Analyte(s)	Aim of study	Endpoint	Sample size	AUC	Study reference
Fetal urine	Peptide signature	Predict postnatal kidney function in patients with LUTO	Kidney function at 2 years	*n* = 66 (*n* = 38 in validation)	0.94	Klein et al., Sci Transl Med 2013 [33]
Fetal urine	Peptide signature	Validation of peptide signature defined in [33] in patients with LUTO	Kidney function at 6 months	*n* = 23	0.94	Buffin-Meyer et al., Pediatr Nephrol 2020 [34]
Fetal urine	Cytokines	Correlation of fetal urine cytokines to β2-microglobulin as a surrogate maker of fetal renal dysfunction	n.a	*n* = 24	n.a	Vieira et al., Pediatr Nephrol 2017 [35]
Fetal urine	Chemokine signature	Predict postnatal kidney function in patients with LUTO	Kidney function at 6 months	*n* = 84 (*n* = 27 in validation)	0.88	Geraud et al., Pediatr Nephrol 2025 [36]
Amniotic fluid	Peptide signature	Predict postnatal kidney function in patients with CAKUT	Kidney function at 2 years	*N* = 178 (51 in validation)	0.96	Klein, Buffin-Meyer et al., Kidney Int 2021 [11]

*n.a*. not applicable

### Posterior urethral valves (PUV)

Lower urinary tract obstruction (LUTO) is the most frequently encountered severe bilateral fetal presentation in patients with CAKUT [37] and is often associated with PUV [5]. Exclusively found in males, PUV is characterized by a valve type obstruction to the outflow of urine from the bladder. This blockage leads to deleterious effects on the urinary bladder and the upper urinary tracts, and is diagnosed in utero with the prenatal ultrasound appearance of a megabladder or megacystis. Specific diagnostic features that can guide clinical approach and decisions are lacking [5].

Currently, PUV is one of the rare CAKUT where prenatal intervention is possible, focusing on decompression of the bladder by either vesico-amniotic shunt (VAS) or fetal endoscopic valve ablation. As previously suggested [38] and described in a recent prospective observational study, VAS procedure during the first trimester is associated with survival and predominantly normal or mildly impaired kidney function in the first year of life [39]. However, a significant proportion of fetuses with LUTO is still diagnosed only in the second trimester, and, unfortunately, neonatal survival after second-trimester VAS did not convincingly improve [40]. This suggests that VAS was performed in non-selected patients to be beneficial, thereby highlighting the need to find reliable prognostic indicators to select cases that will benefit from VAS insertion. Research has therefore focused on means to select, prenatally, fetuses with PUV with most likely normal or mildly impaired postnatal renal function where VAS or fetal endoscopic valve ablation can be useful to avoid further degradation of fetal renal function and avoiding pulmonary hypoplasia [41]. Ruano et al., [42] have proposed such a score based on ultrasound and fetal urine electrolytes, but this score remains to be validated, especially in the light of meta studies showing the absence of predictive value of ultrasound and fetal urine biochemistry used for over several decades [43].

The absence of progress in the personalized selection of fetuses with PUV that would truly benefit from fetal intervention sparked the interest to explore fetal urinary peptide signatures as potential biomarkers predictive of postnatal function in fetuses with PUV. In 28 patients with PUV, a signature of 26 peptides, among 4000 fetal urinary peptides analyzed by MS, was found to be strongly associated with 2-year postnatal renal outcome [33]. This signature, mathematically reduced to 12 peptides (12PUV), showed very good performance in predicting the postnatal onset of early CKD 5 in a small cohort of 38 new and blindly assessed fetuses with PUV. The signature displayed a sensitivity and specificity of 88% and 95%, respectively, associated with an area under the receiver operator characteristic curve (AUC) of 0.94. It also outperformed the conventional parameters including ultrasound-measured characteristics of the fetal kidneys (hyperechogenicity, dysplasia, presence of cysts, or aspect of the renal cortex) and AF volume (oligohydramnios or anhydramnios) as well as fetal urine biochemistry (sodium, β2-microglobulin). Although the validation was performed on a small number of subjects, the results of this study showed for the first time the added value of a peptide-based signature in prenatal medicine compared to conventional models. This fetal urine peptide signature was validated in a fully independent, single center study involving 23 PUV pregnancies from Poland (temporal and geographical validation) with a comparable AUC of 0.94. However, follow-up was limited to 6 months of age compared to the 2-year postnatal end-point in the original study [34]. This small-scale study revealed an additional interesting aspect since it included not only 23 patients with PUV, but also additional eight patients with a megabladder where PUV was not confirmed at birth. Although the 12PUV signature was initially only designed for PUV, it displayed excellent predictive capacity in all patients with a megabladder, irrespective of the presence of PUV (AUC 0.89), thereby strengthening its clinical utility. This might be explained by the fact that the 12PUV peptide signature, for the major part composed of collagen fragments, is probably not representing the molecular signature of the bladder obstruction, but more likely the molecular changes due to tissue remodeling in the kidney as exemplified by the presence of dys/hypoplasia, cysts, etc., in LUTO which determines the postnatal renal outcome [34].

The peptide markers thus appear to primarily reflect tissue remodeling. However, capturing complementary pathways, such as inflammation, which is also known to play a role in the pathophysiology of obstructive uropathies [44], could provide additional, orthogonal information and warrants evaluation of its potential added value. Low-abundance mediators such as inflammatory and profibrotic cytokines, are typically present in the pg/mL range in body fluids including urine [45, 46]. They are less accessible with high throughput-based peptidome analysis which is inherently biased toward relatively abundant urinary peptides. Integrating CE-MS–based peptidomics with targeted assays such as ELISA, which are better suited for low-abundance proteins and peptides, may therefore provide a more comprehensive molecular assessment and improve risk stratification and prediction of postnatal renal outcome.

Using ELISA, Vieira et al., observed increased abundance of many inflammatory proteins (including IL-2, IL-4, IL-6, TNFα, sTNFRI, sTNFRII, IFN-γ, MCP-1/CCL2, eotaxin/CCL11, and IL-8/CXCL8) in urine of 24 fetuses with PUV compared to matched fetal urine of healthy preterm neonates, thereby clearly confirming the inflammatory character of PUV [35]. Most of these inflammatory markers correlated with β2-microglobulin as a surrogate maker of fetal renal dysfunction, but the authors did not correlate the prenatal inflammatory marker data to postnatal renal function outcome which is currently the most important issue in PUV. This was recently examined in a retrospective cohort of 79 PUV patients. Geraud et al., [36] identified the urinary chemokines CCL2 (MCP-1), CXCL9 (MIG), and CCL4 (MIP-1β) as predictors of postnatal kidney failure in a cohort of 57 fetuses with PUV, with validation in an independent cohort (*n* = 27; AUCs 0.87, 0.81, and 0.86, respectively). Although each chemokine performed less well than the previously described 12PUV fetal urine peptide signature [33], their combination showed similar predictive performance.

### “All” CAKUT

**Fig. 3:**
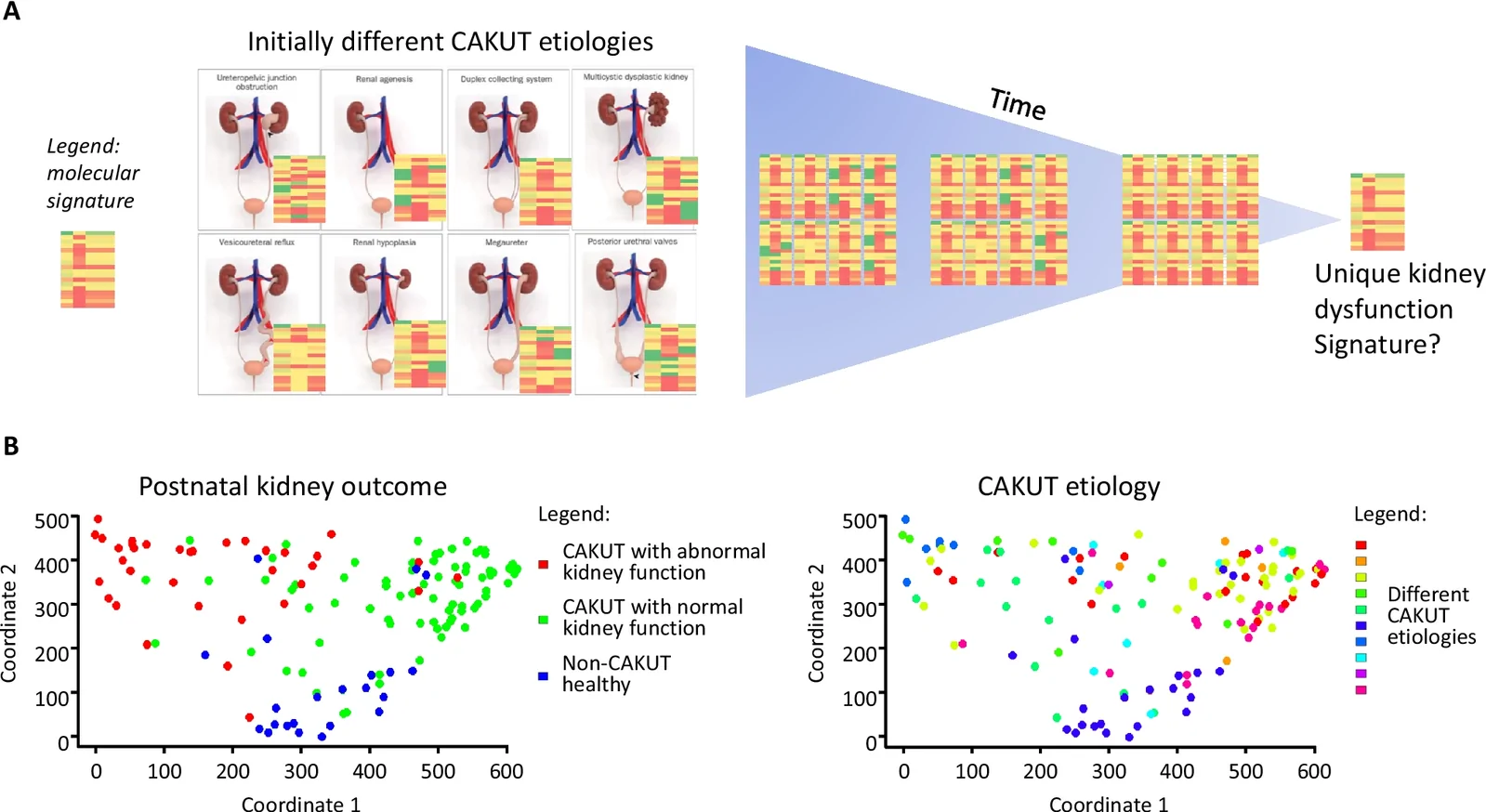
Concept of a shared molecular signature predicting renal outcome in CAKUT. **A** Different prenatal CAKUT etiologies initially present with distinct anatomical abnormalities and molecular patterns. Over time, however, common molecular pathways associated with kidney dysfunction may converge, leading to the emergence of a shared molecular signature that reflects the trajectory toward renal impairment. This concept suggests the possibility of identifying a “one-fit-for-all” biomarker signature predictive of postnatal renal outcome, independent of the initial prenatal etiology. **B** Example from a prospective cohort of prenatal CAKUT cases showing clustering of AF peptidomic profiles. Peptide signatures separate patients more strongly according to postnatal renal outcome than according to the specific CAKUT etiology, supporting the existence of a common molecular signature associated with kidney dysfunction. Parts from Fig. 3A were taken from Nicoleau et al., Nat Rev Nephrol 2015, with permission; Fig. 3B was taken and adapted from Klein, Buffin-Meyer et al., Kidney International, 2021 [11], with permission

The above studies demonstrated that peptides-based markers have potential for PUV. But due to the rarity of the remaining individual bilateral CAKUT etiologies, it is currently impossible to generate prenatal predictive signatures of postnatal renal function for each individual CAKUT etiology. The above studies also suggest that, at relatively late timepoints in nephrogenesis, future renal function could become largely independent of the underlying etiology in LUTO [34]. This opens up the concept for the identification of a “one-fit-for-all” molecular kidney dysfunction signature potentially independent of the initial prenatal etiology (i.e., ultrasound appearance), but strongly associated with, and predictive of, postnatal renal outcome in CAKUT (Fig. 3A). This global molecular change in CAKUT could be compared to the development of fibrosis that is a shared mechanism of CKD progression in many etiologies of kidney diseases, occurring early and conditioning the track to overt CKD [47].

The one-fit-for-all concept was explored by analyzing the AF peptidome as a proxy for the fetal urine peptidome [48] of a large (*n* = 178) prenatal prospective CAKUT cohort associated with a 2-year postnatal follow-up (Clinicaltrials.gov: NCT02675686) [11]). This cohort contained nine different antenatal bilateral CAKUT etiologies, comparable to what was encountered in other, but retrospective, studies including ~ 100 patients. Hierarchical clustering analysis of the AF peptidome data showed that 1008 AF peptides separated CAKUT patients better based on postnatal renal outcome than on the underlying CAKUT etiology (Fig. 3B) supporting the potential existence of a one-fit-for-all AF peptides signature for CAKUT outcome. This study may be biased by the gestational age at which the amniotic fluid sample was collected. Indeed in this cohort, sampling was performed at approximately 25 weeks [11], a relatively late stage where shared molecular mechanisms of CKD progression are likely already established, across different CAKUT etiologies and analogous to the convergence observed in fibrosis pathways in CKD [47]. However, even at this advanced stage, the AF peptidome or proteome retains its ability to capture the etiological differences in CAKUT. For instance, distinct AF proteomic profiles can be identified according to the presence of cysts (enriched for pathways related to programmed cell death and keratinization) or dysplasia (enriched for pathways related to extracellular matrix modification and cell-to-cell communication), although their association with postnatal renal functional outcomes has not been evaluated [12].

Subsequent statistical comparison of the AF peptidome of 35 CAKUT patients with normal outcome (eGFR > 60 ml/min/1.73 m^2^ at age 2 years) and 18 patients with compromised outcome (early-onset renal failure, perinatal death due to CKD 5, or TOP with fetopathology showing severe renal maldevelopment) identified a signature of 98 AF peptides. This signature (bCAKUTPep) was validated in a blinded holdout set of 51 CAKUT patients and predicted postnatal outcome with an AUC of 0.96, an 88% sensitivity, and a 97% specificity, confirming the existence of a one-fit-for-all signature for CAKUT postnatal renal function. Combination of the peptides with AF volume, gestational age, or both, did not significantly improve the AUC. This AF predictive signature outperformed currently used clinical parameters including AF volume (i.e., presence of oligohydramnios/anhydramnios) considered the best clinical predictor of postnatal renal outcome. The AF predictive signature was additionally validated through: (1) a small-scale geographical validation (*n* = 12), and (2) a domain validation using AF from patients with primary maternal cytomegalovirus infection (*n* = 47) associated with neurological lesions of varying severity and healthy fetuses (*n* = 22) [11].

Collagen peptides represented the majority (88%) of the AF peptides that could be potentially linked, similar to 12PUV, to connective tissue turnover. However, the bCAKUTPep signature also included inflammation (osteopontin, inter α trypsin inhibitor heavy chain H4) peptide markers, validating involvement of inflammatory processes as observed in PUV [35]. Three peptide fragments of thymosin-β4, a small protein with known repair and regenerative capacities, showed the strongest association to the severity of CAKUT after the collagen peptides. Knockout of thymosin-β4 modifies to a moderate extent the zebrafish pronephros [11]. In addition, although thymosin-β4 knockout mice do not have a detectable adult renal phenotype, they are sensitized for renal insults while, conversely, administration of exogenous thymosin β4 improves animal models of unilateral ureteral obstruction and diabetic nephropathy [49]. It is therefore possible that modified thymosin-β4 abundance in CAKUT, although without major impact on early renal development, makes the kidney hypersensitive to environmental factors during pregnancy (pre-gestational maternal diabetes mellitus, maternal overweight/obesity, and drug use [1]) typically associated with CAKUT. However, this remains to be studied in more detail.

## Implementation challenge

The major final goal of body fluid-based protein biomarkers is their use in clinical practice as predictors of progressive disease. Until now, ELISA-based strategies have not provided proteins or protein signatures with predictive value for CAKUT, or at least have not been further tested for this capacity. Therefore, their use in routine management of CAKUT appears currently to be compromised. This is different for the fetal urinary and AF signatures that have clearly shown predictive capacities outperforming ultrasound or biochemistry.

As alluded to by several authors, especially in the prenatal stage, definite prognostic markers for postnatal overall survival and the degree of renal impairment would be of immense help in counselling and decision-making [37, 37, 50]. This lack of efficient prognostic methods leads to documented situations in which cases of severe bilateral CAKUT for whom TOP was considered but not performed had normal postnatal renal function, or continuations of pregnancies resulting in early CKD 5 where dialysis, transplantation, or palliative care were not adequately anticipated. The fetal urinary and AF signatures could provide this near definite prognosis and provide clear-cut help in counselling and decision-making. However, routine clinical implementation of these signatures is also not straightforward since several obstacles appear to block a rapid implementation in the clinic.

### Need for prenatal body fluids

To be reliable, such protein-based body fluid signatures need additional prospective independent, preferably, multicenter validation studies [51]. Unlike initiatives for the identification of CAKUT-associated gene variants and deletions that can use blood samples from any point in time allowing to include relatively easily several hundreds of patients, obtaining prenatal protein-based body fluid signatures requires tedious planning and sample storage. This observation together with the rareness of CAKUT, even with many participating centers, makes such validation studies a long-term endeavor. For example, the ANTENATAL study focusing on the validation of the 12PUV fetal urinary peptide signature in patients with PUV started in 2017 with the aim to prospectively include 400 patients from multiple European centers and was planned to last 6.5 years [52] and, due to the Covid epidemic, will only stop in 2026 with a total of ~ 200 inclusions.

### Budgetary constraints

In addition, especially in CAKUT where the major current goal is counselling and decision-making, there is no interest for Pharma-based sponsoring. Therefore, most, if not all, studies focusing on CAKUT are investigator-driven studies with a limited budget. Long-term academic support for such international validation studies is absent. For example, in Europe, the rare-disease program (EJP-RD or more recently ERDERA) and ERA Permed (personalized medicine) only fund 3-year projects, and the NIH rarely funds projects over 4 years. The European commission is financing rare disease networks for longer periods (e.g., ERKNet, www.erknet.org) but this initiative does not fund long-term clinical trials. Clearly, this absence of support for putting in place the complicated logistics for long-term studies at the international level in rare disease is a major obstacle for multicenter validation.

### Regulatory issues

Absence for such support for rare diseases focusing on medical devices including in vitro diagnostic devices was recognized by the FDA in 2016 and identified the lack of biomarkers that more closely mirror disease severity and response to therapy (https://www.fda.gov/media/111315/download). This was also recognized by the international rare disease research consortium (IRDiRC) by creating a specific Diagnostics Scientific Committee (DSC). Although these initiatives are almost entirely focusing on the development of gene-based diagnostics, it raises the hope for the more ready acceptance and support for “orphan” diagnostics also based on non-genetic markers.

### Implementation of mass spectrometry (MS)-based diagnostics

Assessment of the peptide signatures will remain most likely MS-based and hence will need the use of MS in the clinical laboratory. Although MS has made significant contributions to doping/drug testing, rapid identification of microbes, and assessment of immunosuppressant metabolites [53], MS-based assays focusing on proteins or peptides are rare in the routine clinical laboratory.

At first, there appear substantial challenges involved in implementing an MS-based service, including initial high costs of equipment and skilled labor.

#### Cost

It can be reasonably anticipated that, over time, the initial capital investment and equipment maintenance costs will be offset by the recurring reagent costs associated with antibody-based assays. Cost will also be reduced when using the same equipment for multiple MS-based assays. For example, analysis of urinary peptides biomarkers can be applied using the same equipment for CAKUT [11], prediction of diabetic kidney disease in type 2 diabetic individuals [54], and presence of significant prostate cancer [55]. For drug detection, MS-based strategies in the clinical laboratory have been shown to be cost-effective already almost 2 decades ago.

#### Labor

Skilled labor is important but with the establishment of standard operating protocols; the analysis of urinary peptides by capillary electrophoresis coupled to MS (CE-MS) is highly reproducible between different laboratories [56]. Quality control analysis including preparation stability and short- and long-term precision also showed low technical variability, below the anticipated biological variability for both urinary and AF-peptides [11]. Any well-trained engineer can thus perform such analyses. These different characteristics of MS-based analysis of urinary peptides has led already at an early stage to a letter of support of the FDA for an adult CKD signature, but full regulatory approval is still not obtained for this signature [57]. Again, there is hope that the rare disease status of CAKUT might help to speed up the clinical implementation of such MS-based strategies.

## Outlook

Due to routine ultrasound screening during pregnancy, CAKUT is nowadays detected at an early stage. Furthermore, the underlying genetic causes of CAKUT are increasingly well defined. However, information on disease severity and progression is still chronically lacking, hampering optimal management of bilateral CAKUT. Above, we have seen that the use of prenatal body fluid peptide signatures could improve this *status quo* in the short term, but hurdles still remain for routine implementation. In addition, in our opinion, the strong link between inflammation markers of CAKUT and disease progression has not been adequately explored. This also points to a general shortcoming in CAKUT biomarker research: very often studies only compare association of body fluid markers to the presence or absence of CAKUT which has little clinical relevance with respect to the management of the disease. We recognize that prenatal body fluid markers are accompanied by issues related to sampling, and that in many countries or even in different clinics in the same country, there are hesitations with respect to prenatal body fluid sampling. We therefore also advocate to improve imaging. Although MRI will not be routinely available in CAKUT, standardization of ultrasound definitions, such as the recently proposed “kidney sonotype” [8] combined with AI-trained machine learning to link multiple kidney sonotypes to postnatal outcome, should be explored. Finally, additional omics levels [29] and combining those with kidney sonotypes should allow to further improve the description of CAKUT and thus prediction of postnatal renal function.

## Key summary points

1. Prenatal ultrasound detects CAKUT but has limited ability to predict postnatal renal outcome, particularly in bilateral disease.
2. Genetic testing improves etiological classification of CAKUT but provides limited prognostic value due to weak genotype–phenotype correlations.
3. Molecular biomarkers in prenatal body fluids, particularly peptide-based signatures, may better capture disease activity and predict renal outcomes.
4. Proteomic analysis of fetal urine or AF has demonstrated strong prognostic performance, outperforming conventional imaging and biochemical markers.
5. Clinical implementation of peptide-based biomarkers requires large multicenter validation studies but is challenging due to the rarity of CAKUT.

## Multiple choice questions

Answers are given following the references.

1. Which of the following best explains why prenatal ultrasound has limited predictive value for postnatal renal outcome in bilateral CAKUT?
  (A) Ultrasound cannot detect structural kidney anomalies before the third trimester
  (B) Ultrasound only evaluates macroscopic anatomy and does not capture ongoing molecular disease processes
  (C) Ultrasound overestimates AF volume in normal pregnancies
  (D) Ultrasound cannot identify urinary tract obstructions
  (E) Ultrasound measurements are always operator-independent
2. Which of the following genetic findings currently has the strongest evidence as a monogenic cause of CAKUT?
  (A) WT1 and PKD1
  (B) PAX2 and HNF1B
  (C) ACE and AGT
  (D) UMOD and NPHS1
  (E) COL4A5 and LAMB2
3. Why are peptide-based biomarkers often preferred over full-length proteins in urinary or AF proteomic studies?
  (A) Peptides require antibody-based assays rather than mass spectrometry
  (B) Peptides are less stable and degrade quickly after sample collection
  (C) Peptides can be analyzed directly by mass spectrometry without tryptic digestion, improving reproducibility
  (D) Peptides are only detectable after glomerular filtration failure
  (E) Peptides are not affected by renal disease processes
4. Why are multimarker molecular signatures generally preferred over single biomarkers for predicting outcomes in complex diseases such as CAKUT?
  (A) Single biomarkers cannot be measured in body fluids
  (B) Multimarker signatures can capture multiple biological pathways and better reflect disease heterogeneity
  (C) Multimarker signatures are easier to measure than single biomarkers
  (D) Single biomarkers cannot be detected using mass spectrometry
  (E) Multimarker signatures eliminate the need for clinical or imaging data
5. Which of the following represents a major challenge for the clinical implementation of prenatal peptide-based biomarkers in CAKUT?
  (A) The inability of mass spectrometry to detect peptides in biological fluids
  (B) The need for large prospective multicenter validation studies in a rare disease context
  (C) The lack of any reproducibility of mass spectrometry-based peptide measurements
  (D) The impossibility of storing AF samples for proteomic analysis
  (E) The absence of any detectable peptides in fetal urine or AF

## Supplementary information

Below is the link to the electronic supplementary material.

Graphical abstract (PPTX 171 KB)
